# Interaction of APOBEC3A with DNA Assessed by Atomic Force Microscopy

**DOI:** 10.1371/journal.pone.0099354

**Published:** 2014-06-06

**Authors:** Luda S. Shlyakhtenko, Alexander J. Lushnikov, Ming Li, Reuben S. Harris, Yuri L. Lyubchenko

**Affiliations:** 1 Department of Pharmaceutical Sciences, College of Pharmacy, University of Nebraska Medical Center, Omaha, Nebraska, United States of America; 2 Department of Biochemistry, Molecular Biology, and Biophysics, Institute for Molecular Virology, Center for Genome Engineering, Masonic Cancer Center, University of Minnesota, Minneapolis, Minnesota, United States of America; German Cancer Research Center, Germany

## Abstract

The APOBEC3 family of DNA cytosine deaminases functions to block the spread of endogenous retroelements and retroviruses including HIV-1. Potency varies among family members depending on the type of parasitic substrate. APOBEC3A (A3A) is unique among the human enzymes in that it is expressed predominantly in myeloid lineage cell types, it is strongly induced by innate immune agonists such as type 1 interferon, and it has the capacity to accommodate both normal and 5-methyl cytosine nucleobases. Here we apply atomic force microscopy (AFM) to characterize the interaction between A3A and single- and double-stranded DNA using a hybrid DNA approach in which a single-stranded region is flanked by defined length duplexes. AFM image analyses reveal A3A binding to single-stranded DNA, and that this interaction becomes most evident (∼80% complex yield) at high protein-to-DNA ratios (at least 100∶1). A3A is predominantly monomeric when bound to single-stranded DNA, and it is also monomeric in solution at concentrations as high as 50 nM. These properties agree well with recent, biochemical, biophysical, and structural studies. However, these characteristics contrast with those of the related enzyme APOBEC3G, which in similar assays can exist as a monomer but tends to form oligomers in a concentration-dependent manner. These AFM data indicate that A3A has intrinsic biophysical differences that distinguish it from APOBEC3G. The potential relationships between these properties and biological functions in innate immunity are discussed.

## Introduction

Human cells can express up to seven APOBEC3 (A3) proteins, which have the capacity to inhibit a broad number of endogenous and exogenous retroelements including viruses such as HIV-1 [1], [2], [3], [4], [5]. Each A3 protein has one (A3A, A3C, A3H) or two (A3B, A3D, A3F, A3G) zinc-coordinating domains and elicits single-stranded DNA (ssDNA) cytosine deaminase activity [6], [7], [8], [9], [10]. The most intensively studied proteins are A3F and A3G because they potently block the replication of Vif-deficient HIV-1 [4], [11], [12], [13], [14], [15], [16], [17], [18]. In contrast, A3A is not a strong inhibitor of Vif-deficient HIV-1 in T cell lines [18]. However, it may have an antiviral role in myeloid lineage cell types because it is strongly induced by type 1 interferons and other innate immune agonists [2], [8], [10], and it can also trigger the clearance of naked double-stranded DNA (dsDNA) by a deamination and uracil excision-dependent mechanism [10]. Further consistent with a role in innate immunity, A3A has been shown to suppress the replication of LINE-1 [9], AAV-2 [6], and HTLV-1 [3], [8], [9] in model systems.

A3A is arguably the most active deaminase family member in humans [19]. A3A preferentially deaminates 5′-TC dinucleotide within ssDNA [1], [19], binds ssDNA with a micromolar dissociation constant [1], [20], and does not form high molecular mass complexes [9]. A3A has a single H-X-E-X_28_P_–_C-X_4-_C zinc-coordinating catalytic motif [9], [21] and models for ssDNA interaction have been proposed [20], [22]. Mutation of its active site glutamic acid E72 causes a complete loss of both deaminase [9], [10] and antiviral activity [8], [10], [22]. A unique property of A3A is an ability to deaminate 5-methylcytosine (5 mC) into thymine with an efficiency approximately 5- to 10-fold less than its C to U deamination activity [2], [19]. A3A over-expression can trigger genomic DNA instability and a global DNA damage response [19], [23], [24], [25]. Taken together, A3A is a unique member of APOBEC3 family.

Despite recent progress on A3A biochemistry [1], [19], [26] and structural biology [20], limited information is available for biophysical properties such as DNA binding specificity, stoichiometry in complex with DNA, and propensity to exist as a monomer or an oligomer. Here we address these properties by applying atomic force microscopy (AFM) together with a hybrid DNA approach recently validated in studies on A3G [27], [28], [29] and SSB [30]. Our AFM studies revealed that A3A binds ssDNA as a monomer even at a large excess of protein over DNA. Also, free A3A protein remains in a monomeric state in the absence of DNA. This property contrasts with A3G, which has a tendency to oligomerize at concentrations exceeding equimolar ratios [29]. Additionally, A3A has the capacity to bind both single- and double-stranded DNA, whereas A3G will only form complexes with ssDNA. Our studies are consistent with a model for A3A in providing innate immunity to DNA-based parasites.

## Materials and Methods

### Preparation of APOBEC3 Proteins

Recombinant human A3A-mycHis and A3A–E72A-mycHis proteins were purified from 293T cells as described [10], [31]. The purity and concentration of these proteins was assessed by Coomassie Blue R250 staining and densitometry. A3G-191-384 was produced in *E. coli* as a GST fusion and purified as described [31]. See also Fig. S1 in File S1.

### Fluorescence-based DNA Deamination Assay

Fluorescence based ssDNA cytosine deamination assays were performed as previously described [10], [31], [32]. The substrate oligo is 5′-(6-FAM)-AAA-TTC-TAA-TAG-ATA-ATG-TGA-(TAMRA)-3′.

### Preparation of Hybrid DNA Substrate

The hybrid DNA substrate, in which a ssDNA region is flanked by dsDNA arms, was described in detail previously [28], [30], [33]. The final product has a 69 nucleotide ssDNA region in the middle (5′- TAT TAA AGA GAA AGT GAA ACC CAA AGA ATG AAA ACC CAA ATG TTA GAA TTG TTA AGT GAA ACC CAA AGA -3′) flanked by 231 bp and 441 bp of dsDNA.

### Preparation of A3A and A3A_E72A_ Hybrid DNA Complexes

The hybrid DNA was incubated with A3A or A3A_E72A_ at different protein to DNA ratios at 37°C for 10 min. The reaction buffer contained 50 mM HEPES (pH 7.5), 100 mM NaCl, 5 mM Mg^2+^ and 1 mM DTT. All complexes were purified using Montage UFC spin column as described [28].

### Samples Preparation and AFM Imaging

Sample preparation was identical to the protocols described previously [28], [30], [33]. Briefly, ∼5 µL of sample was deposited on the APS mica surface [34] for 2 min., rinsed with deionized water, and dried with Argon gas. Nanoscope IV multimode system from Bruker (Santa Barbara, CA) was used for imaging samples. Regular silicon probes with spring constant ∼42 N/m and resonance frequencies between 310 and 340 kHz were used.

### Data Analysis

The details for data analysis were described previously [28], [30], [33]. Briefly, the Femtoscan Online (Advanced technologies Center, Russia) was used for image analysis of free proteins and protein-DNA complexes. For each protein-DNA complex, Femtoscan software was used to determine protein volume, complex yield, and the length of each dsDNA region adjacent to the intervening ssDNA. The DNA contour length was measured using the Curve-Tool from Femtoscan software. The protein volume was obtained as described in [35] by measuring the height and lateral dimensions of the protein using Cross-Section tool from Femtoscan software. The volume of the protein was transformed into protein molecular weight using the calibration curve provided in [30]. Additional specifics to the methods can be found in Supplementary Data, Section S1. For each type of measurements at least 100 complexes were analyzed and the data were assembled into histograms using Origin 6.0 software (Originlab, MA). Full volume measurement data sets are provided in Supplementary Data, Section S2.

## Results

### 1. Wildtype A3A Properties

**Figure 1:**
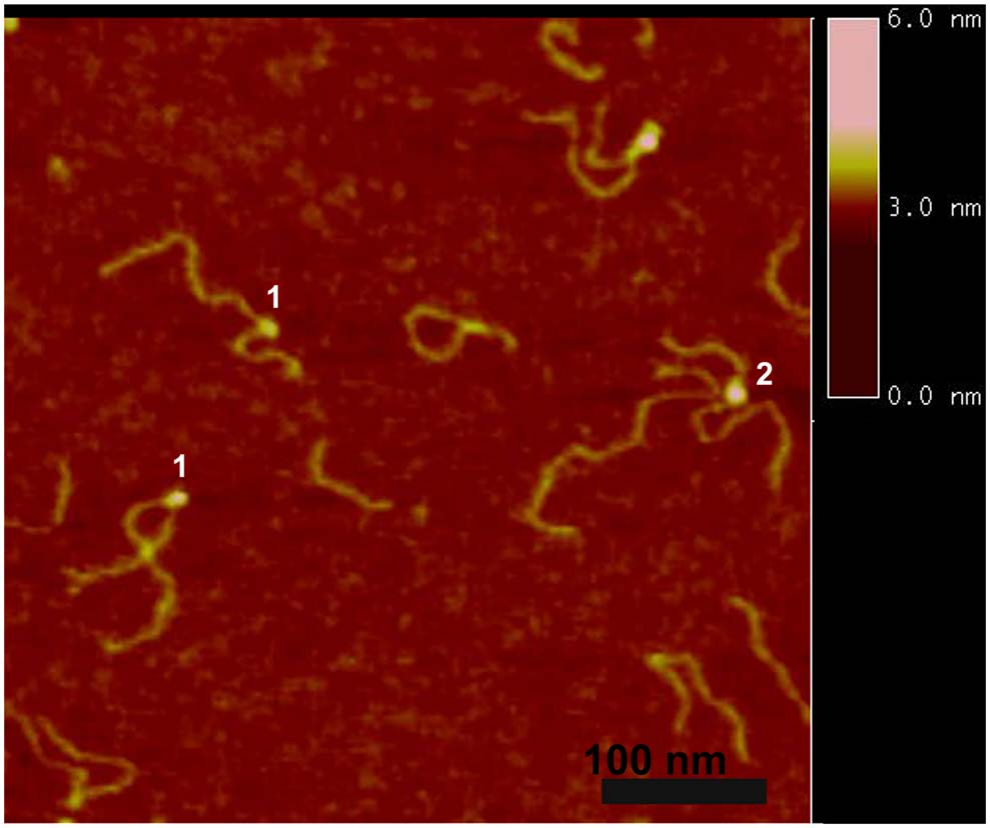
AFM images of A3A in complex with gap-DNA. A3A monomer and dimer complexes are labeled 1 and 2, respectively. Bar size, 100

**Figure 2:**
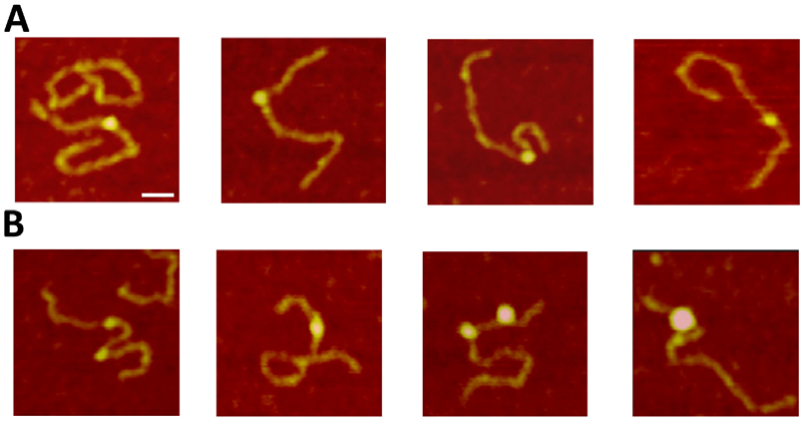
Gallery of AFM images of A3A complexed with ssDNA (A) or dsDNA (B) regions of the gap-DNA substrate. Bar size, 30

We used a DNA substrate with a 69 nucleotide ssDNA region flanked by duplexes (hybrid gap-DNA) to visualize complexes of A3A with DNA. A typical AFM image of A3A gap-DNA complexes is shown in Fig. 1. The protein is seen as a small bright blob located within the DNA substrate at a position corresponding to the location of the intervening ssDNA segment. Measured lengths of the dsDNA flanks confirm the position of the protein on the ssDNA region of the hybrid DNA. Protein blobs of different sizes are clearly seen in Fig. 1, indicating different stoichiometries. Based on volume measurements, A3A monomers and dimers are labeled on the image as 1 and 2, respectively. In contrast to A3G [28], [33], the formation of complexes between A3A and hybrid DNA required a rather high protein/DNA ratio (P/D). For instance, A3A gap-DNA complexes were only detectable with P/D of ≥100. The yield of complexes under these high P/D conditions was ∼80%. Additional images of A3A bound to ssDNA segments are shown as a gallery in Fig. 2A. In addition, we also observed A3A associating with double-stranded regions of the hybrid DNA substrate, as in the second panel of Fig. 2B. These A3A-dsDNA complexes were termed non-specific, because prior studies demonstrated that A3A only deaminates ssDNA cytosines [19] and the yield of these complexes was lower (∼20%).

**Figure 3:**
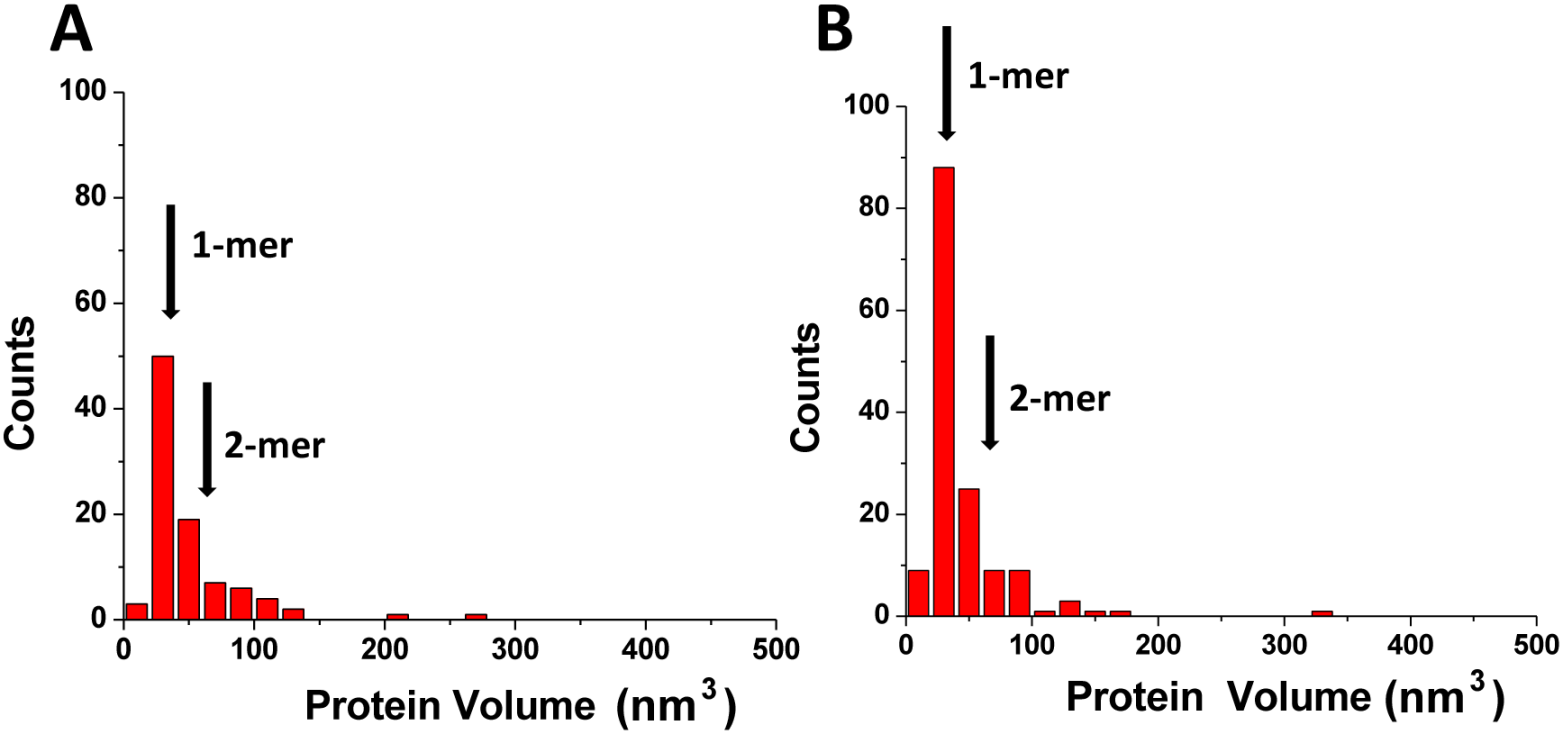
Results of the volume measurements of A3A complexed with gap-DNA (A) alone (B). Numbers of complexes analyzed are N = 118 for (A) and N = 148 for (B). The mean volume values (± SD) for monomers (1-mers; 33±16 nm^3^) and dimers (2-mers; 66±26 nm^3^) are indicated with arrows.

**Figure 4:**
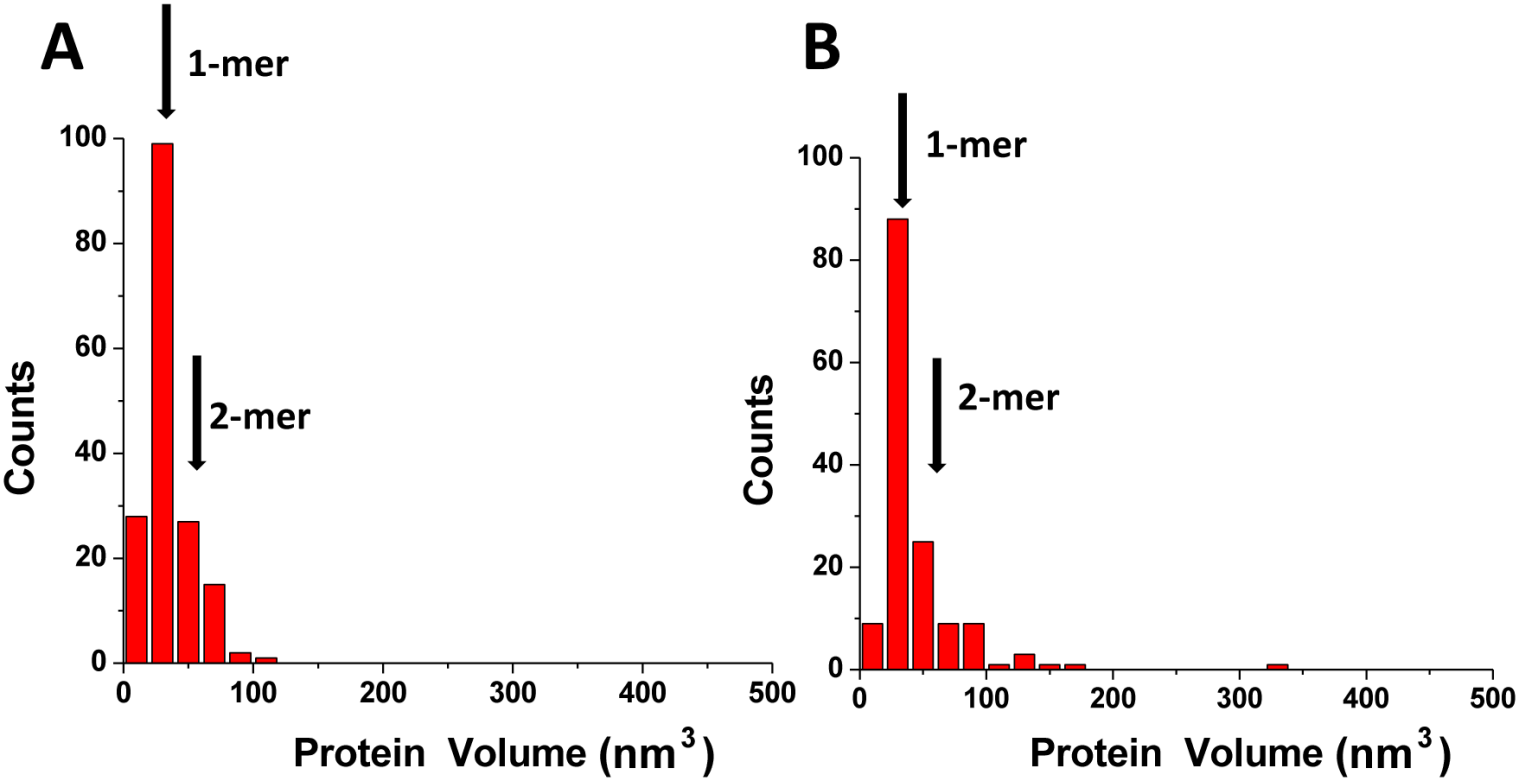
Histograms for protein volume measurements for free A3Gctd (A) and A3A (B). Number of complexes analyzed were N = 173 and 148 for histograms (A) and (B), respectively. The mean volume values for monomers (1-mers; 28±12 nm^3^) and dimers (2-mers; 56±22 nm^3^) are indicated with arrows. See similar numbers for A3A in Fig. 3.

We next performed a statistical analysis of dozens AFM images for each condition to determine the stoichiometry of A3A bound to gap-DNA. These data are shown as a histogram in Fig. 3A. The distribution is rather narrow with the maximum corresponding to the monomeric size of A3A with a relatively small number of dimers and higher oligomers. To determine the protein stoichiometry in the absence of DNA, we imaged A3A alone and the volume distribution for free protein is shown on Fig. 3B. This distribution is very similar to the one for the complex indicating that the monomer is predominate state for free A3A as well as for protein bound to ssDNA. This finding is consistent with results obtained in [9] where it was shown in solution that A3A exists as monomers or small oligomers. Altogether our AFM studies show that A3A exists predominately as a monomer, and it remains monomeric when bound to DNA. This property of A3A is very different from that of A3G, which has a strong propensity for oligomerization [29]. So, because A3A is more similar to A3Gctd than A3Gntd, one may assume that the N-terminal DNA binding domain of A3G is involved in oligomerization. To test this hypothesis, we analyzed the oligomerization state of the A3G catalytic domain A3G-191-384-2K3A (A3Gctd, [31]) by AFM. The histogram distributions for A3Gctd and A3A obtained in the same range of proteins concentrations are shown in Figs. 4A and B, respectively. Both distributions are rather narrow with maxima corresponding to the monomeric state of each protein. This finding is in line with the NMR results for A3A [20] and A3Gctd [36], [37] and the crystal structures of A3Gctd [36], [38], [39].

Taking further advantage of our hybrid DNA approach [28], [30], [33], we were able to simultaneously evaluate A3A’s propensity to bind both ssDNA and dsDNA. As a result we revealed that A3A also associates with dsDNA (Fig. 2, panel B). This is the first direct evidence for A3A binding to such DNA substrate. The yield of such complexes is relatively high although less than the complexes with ssDNA suggesting that ssDNA is still the preferred substrate.

### 2. A3A Catalytic Mutant Properties

**Figure 5:**
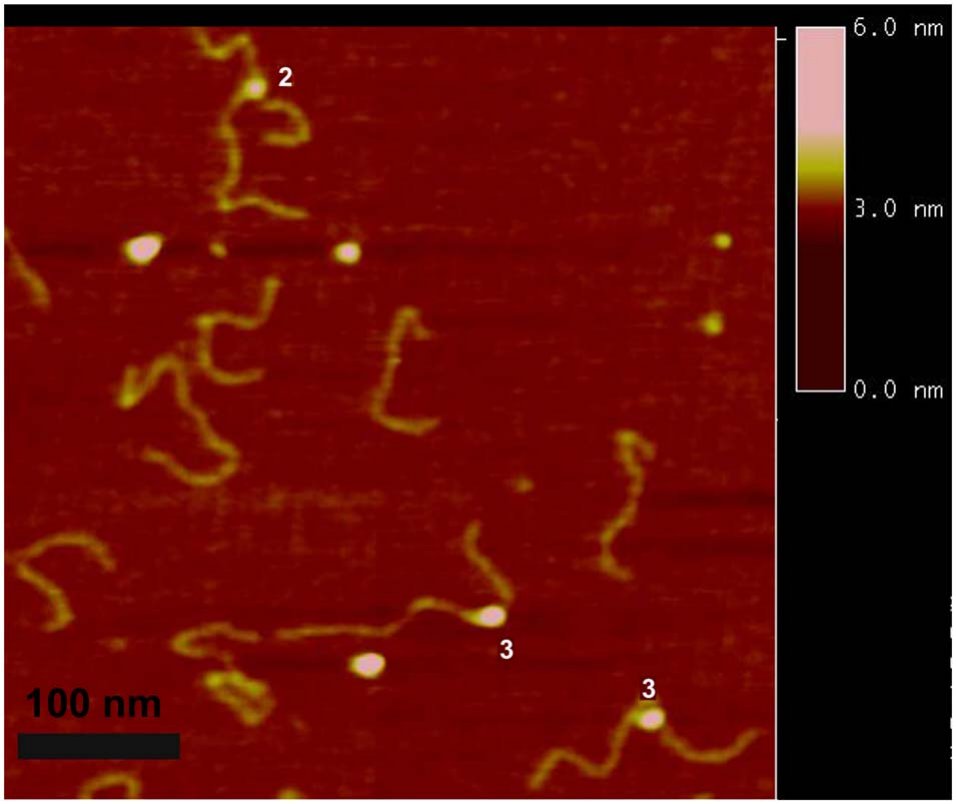
AFM images of A3A_E72A_ complexed with gap-DNA substrate. Specific complexes with A3A_E72A_ dimers and trimers are labeled 2 and 3, respectively. Bar size, 100 nm.

**Figure 6:**
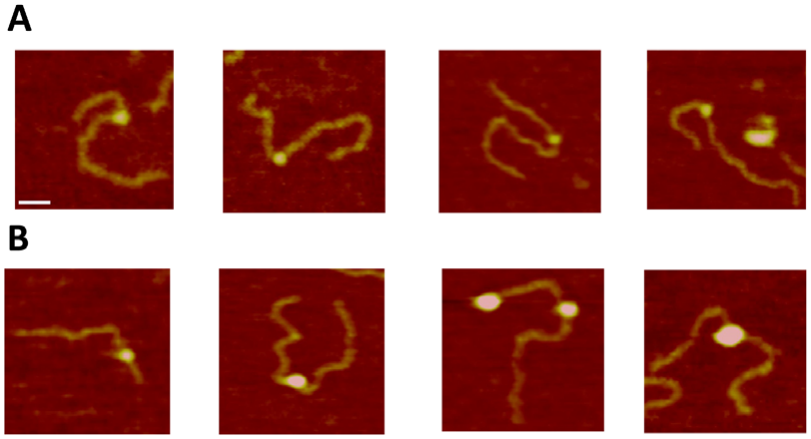
Gallery of AFM images of A3A_E72A_ complexed with dsDNA (A) or ssDNA (B) regions of the gap-DNA substrate. Bar size, 30

**Figure 7:**
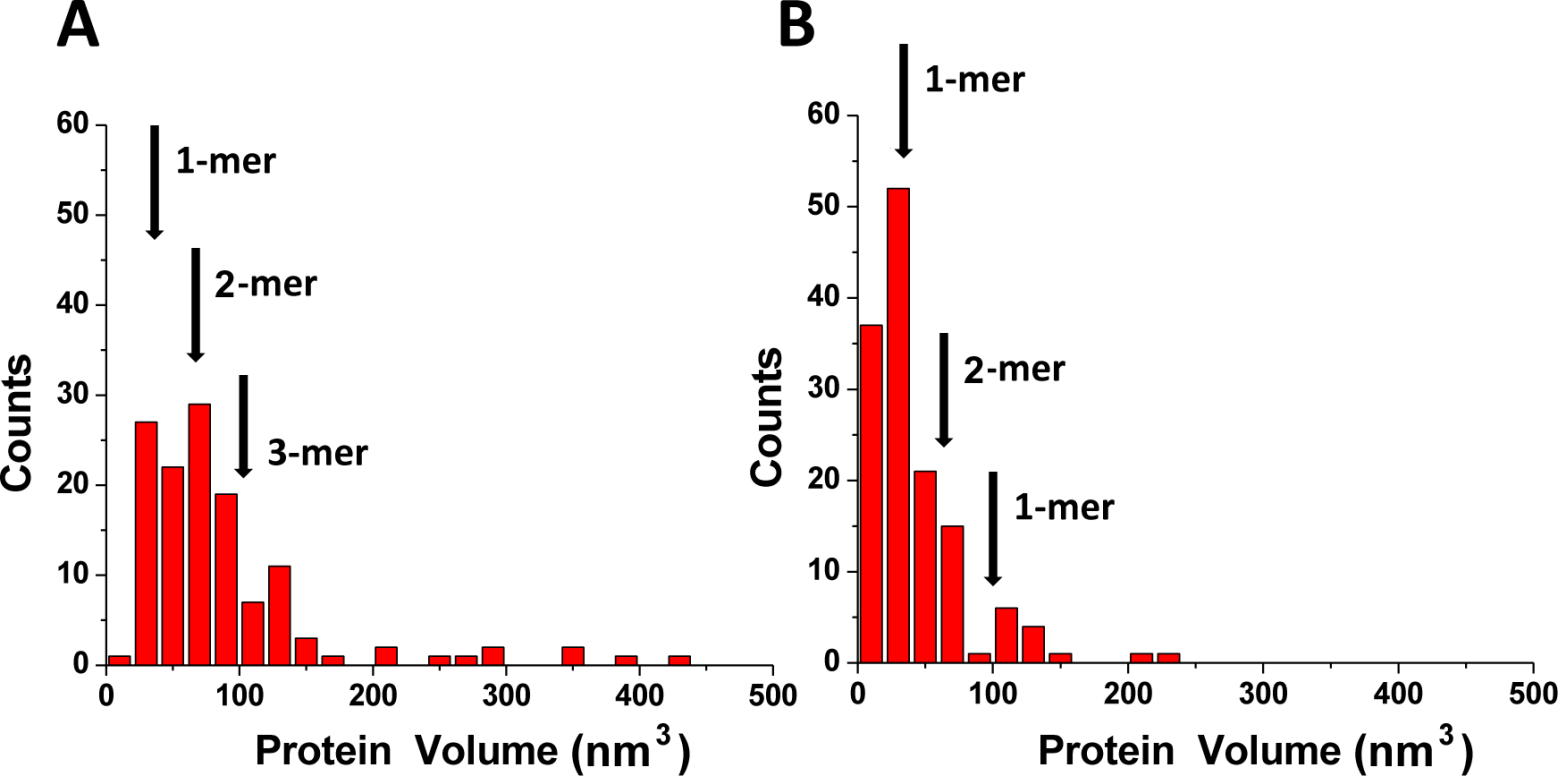
Volume measurements for A3A_E72A_ complexed with gap-DNA (A) and alone (B). Number of complexes analyzed were 167 and 140 for histograms (A) and (B) respectively. The mean volume values for monomers (1-mers; 33±14 nm^3^), dimers (2-mers; 66±24 nm^3^) and trimers (3-mers; 99±34 nm3) are indicated with arrows.

According to a recent NMR study of A3A-ssDNA complexes, the DNA substrate occupies an extended surface on the protein [20]. To test how the catalytic activity of A3A affects its binding efficiency, we examined the properties of A3A_E72A_, which is catalytically defective [8], [19], [40]. A typical AFM image of the complex between A3A_E72A_ and gap-DNA is shown on Fig. 5. Similar to A3A, the catalytic mutant was capable of forming complexes with hybrid DNA also at high protein-to-DNA ratios with about the same yield (∼78%). Based on the estimation of the protein volume for A3A_E72A_ protein we labeled dimers as 2 and trimers as 3 on the AFM image, respectively. The gallery of different complexes of A3A_E72A_ with hybrid DNA is shown in Fig. 6 A, B, where various binding modes and sizes of the protein are indicated in separate frames. Similar to the wildtype protein, A3A_E72A_ binds dsDNA with relatively high efficiency (∼21%) to dsDNA (frames in panel B) but, in contrast to the wildtype protein that remains monomeric upon DNA binding, the A3A_E72A_ mutant forms blobs of different sizes in complex with DNA. The stoichiometry of A3A_E72A_ with gap-DNA is illustrated by volume measurements in Fig. 7A. The distribution is broad with almost equal presence of monomers and dimers with a visible amount of trimers and larger oligomers. In parallel, we analyzed the stoichiometry of the free protein. The results of these volume measurements are shown in Fig. 7B. The volume distribution is narrow with the maximum near the size of wildtype A3A monomers. Therefore, similar to wildtype A3A, free A3A_E72A_ exists in solution mostly as a monomer but, in contrast to wildtype A3A, the mutant has a greater propensity to form oligomers in DNA complexes.

## Discussion

The human APOBEC3 proteins are widely accepted inhibitors of retroviruses and retrotranspons. The single-domain A3A protein has many activities: degrades foreign DNA, blocks replication of the exogenous viruses, and deaminates both normal and 5-methylcytosine. Abundant information of the biological activity of A3A has been reported but rather limited biophysical and structural data are available. In this study we evaluated the DNA binding properties of A3A protein using AFM. The hybrid DNA approach developed in our previous publications allow us directly observe the binding of A3A protein not only with ssDNA, but also with dsDNA. We found that in ∼20% cases A3A protein binds dsDNA, which is the first direct visualization of such DNA-binding activity. A3A overexpression can lead to a global DNA damage response and genotoxicity [25], [41], [42]. We hypothesize that binding of catalytically active A3A to dsDNA may play role in this cascade of events by, for instance, helping the enzyme scan DNA for single-stranded gaps. This dsDNA binding activity also may play role in the restriction of foreign DNA [19]. The binding of dsDNA was particularly clear for catalytically defective A3A_E72A,_ which suggests that substitution of Glu72 with Ala regardless of the drop of the deaminase activity does not affect binding to the substrate, even increasing the protein binding efficiency to dsDNA. At the same time, this substitution changes the oligomerization properties of A3A. Indeed, both wildtype and A3A_E72A_ exist in solution mostly as monomers (Fig. 3B and 7B, respectively), but A3A_E72A_ has a greater tendency to multimerize when bound to DNA (Fig. 6). These data suggest that Glu72 may somehow prevent A3A oligomerization, whereas the substitution of Glu72 with Ala stabilizes oligomers but only in complexes with ssDNA.

As discussed above, A3A is capable of binding dsDNA. Such an activity was not observed for A3G under identical AFM conditions using the same hybrid DNA substrate. Moreover, the A3G assembly into oligomers does not change the strong specificity of A3G to ssDNA substrate. The ability of A3A to bind dsDNA may help to explain the A3A deamination of papillomavirus and plasmid DNA [43]. However, we cannot exclude that the deamination occurs on transiently opened (looped) segments of the DNA duplex as proposed [22].

Another important finding of this study is the fact that a high concentration A3A is needed for the formation of the A3A-hybrid DNA complexes. This suggests a rather transient interaction between A3A and DNA, which is in line with the micromolar range of A3A dissociation constants [1], [20], [22]. In comparison, A3G forms much more stable complexes with ssDNA with dissociation constants almost two orders of magnitude lower [29], [44]. The high stability of A3G-ssDNA complexes can be explained by the contribution of the positively charged N-terminal DNA binding domain [44]. Indeed, our AFM data with A3Gctd support this hypothesis. A3Gctd exists in solution as monomer (Fig. 4A, B) and also required high concentrations for formation of DNA complexes.

The low A3A DNA binding propensity is at odds with its high deamination activity. A3A deamination activity is ∼200 times higher than that of A3G [19]. To reconcile the low DNA binding activity of A3A with its elevated enzymatic activity we propose that A3A uses a “hit and run” mechanism by performing the deamination reaction in a weak complex with ssDNA. This model suggests that deamination activity does not require strong binding with the DNA substrate, so the deamination reaction *per se* can be rapid and occur in the context of transiently formed complexes. If the deamination reaction for A3G is as rapid as that of A3A, then the elevated antiviral activity of A3G may be explained by much stronger ssDNA binding activity, which may prevent the translocation of the viral replication machinery [45], [46].

Finally, A3A exists in solution as a monomer regardless of protein concentration. It retains this monomeric state in complexes with ssDNA. In contrast, A3G forms oligomers in the concentration dependent manner. These findings are in line with a recent paper in which the oligomerization of A3A and A3G was studied using molecular brightness analysis [47]. A3Gctd alone, like A3A, does not aggregate suggesting that the aggregation property of full-length A3G is mediated by its N-terminal domain. This model is in line with the recent comparative analysis of A3A and A3G structures [22]. Our studies are consistent with a model for A3A in providing innate immunity to foreign DNA as well as DNA-based parasites.

## Supporting Information

File S1 Section 1– Purification procedure for Recombinant human A3A-mycHis and A3A-E72A-mycHis proteins. Section 2 - Measurements of protein stoichiometry with AFM. Section 3 - The datasets used for the calculation of the protein stoichiometry with AFM.
(PDF)
