# Supplementary material for: Interaction of APOBEC3A with DNA Assessed by Atomic Force Microscopy
Source: PLoS One. 2014 Jun 6;9(6):e99354. doi: 10.1371/journal.pone.0099354 (PMC4048275; doi:10.1371/journal.pone.0099354)
Supplement: File S1 — Section 1– Purification procedure for Recombinant human A3A-mycHis and A3A-E72A-mycHis proteins. Section 2 - Measurements of protein stoichiometry with AFM. Section 3 - The datasets used for the calculation of the protein stoichiometry with AFM. (PDF) [file pone.0099354.s001.pdf]

# Supplementary Information

***Section S1***.
Recombinant human A3A-mycHis and A3A-E72A-mycHis proteins were purified from 293T cells as described [1, 2]**.** The purity and concentration of these proteins was assessed by Coomassie Blue R250 staining and densitometry **(**see **Figure S1).**

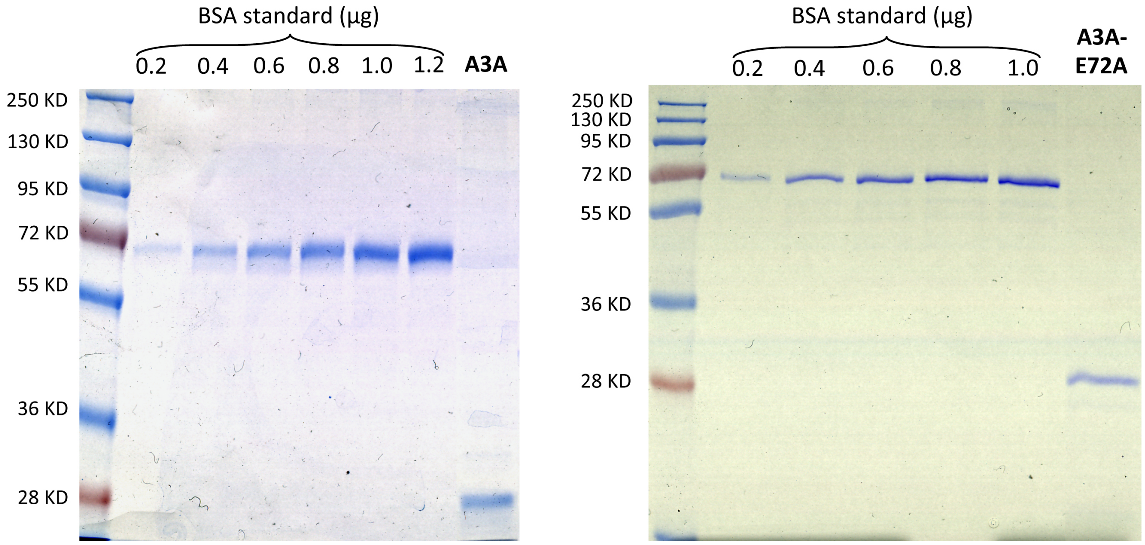


**Figure S1.** Image of A3A-mycHis and A3A-E72A-mycHis proteins separated by SDS-PAGE and stained with Coomassie Blue R250 (left panel). A titration of BSA was used for quantification (right panel).

***Section S2. Measurements of protein stoichiometry with AFM***

The size of the protein in the complex was obtained from the volume data enabling the conversion of the volume measurements of the AFM images of proteins to their molecular weights [3, 4]. In the volume measurements, the protein was approximated as a segment of a sphere using equation S1 derived from reference [3]:

$$V = \frac{h\pi}{6}\left(\frac{3d}{4} + h^2\right) \qquad \text{(eq. S1)}$$

In this equation $V$ is the protein volume, $h$ is the height of the protein measured by the difference in intensity of the protein compared to the background noise by performing cross sections at orthogonal directions. Diameter *(d)* is the widths measured at half of the maximal protein height. Lateral dimensions of the AFM images of macromolecules depend on the tip geometry; therefore the volume of the protein was converted into mass in kilodaltons using a calibration curve generated by the AFM volume measurements of a set of globular proteins with known molecular weights [4]. Lateral dimensions of DNA on AFM images of protein-DNA complexes were used as internal standards for combining data obtained in different scans.

This approach for the volume measurements was used in our early publications

including the AFM study of APOBEC3 proteins [5-8].

***References***


1. Stenglein, M. D., Burns M. B., Li M., Lengyel J., Harris R. S. (2010) APOBEC3 proteins mediate the clearance of foreign DNA from human cells. *Nature structural & molecular biology* **17**: 222-229.
2. Shlyakhtenko, L. S., Lushnikov A. Y., Miyagi A., Li M., Harris R. S., Lyubchenko Y. L. (2012) Nanoscale structure and dynamics of ABOBEC3G complexes with single-stranded DNA. *Biochemistry* **51**: 6432-6440.
3. Henderson, R. M., Schneider S., Li Q., Hornby D., White S. J., Oberleithner H. (1996) Imaging ROMK1 inwardly rectifying ATP-sensitive K+ channel protein using atomic force microscopy. *Proceedings of the National Academy of Sciences of the United States of America* **93**: 8756-8760.
4. Ratcliff, G. C., Erie D. A. (2001) A novel single-molecule study to determine protein--protein association constants. *Journal of the American Chemical Society* **123**: 5632-5635.
5. Shlyakhtenko, L. S., Gilmore J., Portillo A., Tamulaitis G., Siksnys V., Lyubchenko Y. L. (2007) Direct visualization of the EcoRII-DNA triple synaptic complex by atomic force microscopy. *Biochemistry* **46**: 11128-11136.
6. Shlyakhtenko, L. S., Lushnikov A. Y., Li M., Lackey L., Harris R. S., Lyubchenko Y. L. (2011) Atomic force microscopy studies provide direct evidence for dimerization of the HIV restriction factor APOBEC3G. *J Biol Chem* **286**: 3387-3395.
7. Shlyakhtenko, L. S., Lushnikov A. Y., Miyagi A., Li M., Harris R. S., Lyubchenko Y. L. (2013) Atomic force microscopy studies of APOBEC3G oligomerization and dynamics. *Journal of structural biology* **184**: 217-225.
8. Shlyakhtenko, L. S., Lushnikov A. Y., Miyagi A., Lyubchenko Y. L. (2012) Specificity of binding of single-stranded DNA-binding protein to its target. *Biochemistry* **51**: 1500-1509.


***Section S3. The datasets of the protein stoichiometry with AFM***

Each dataset (column) contains the results of several independent experiments allowing us to collect not less than 100 data points; the figure number corresponding to the table is indicated.

| A3G-CTD- protein volume ( Fig.4A), N=173 | A3A-complex-volume (Fig. 3A), N=118 |
| --- | --- |
| 41.84351 | 25.70195 |
| 41.21562 | 25.70195 |
| 73.25616 | 45.48395 |
| 25.89926 | -- |
| 19.10866 | 43.87156 |
| 30.4017 | 51.24951 |
| 23.93034 | 53.33395 |
| 31.26051 | 48.01583 |
| 26.70255 | 57.04333 |
| 25.09419 | 28.32856 |
| 38.47136 | 25.64333 |
| 22.95666 | 70.91795 |
| 26.97722 | 20.36395 |
| 30.60494 | 57.04333 |
| 28.58166 | 57.04333 |
| 17.5847 | 57.04333 |
| 18.45435 | 38.26195 |
| 23.34709 | 31.66795 |
| 38.95307 | 25.70195 |
| 27.95852 | 34.38993 |
| 39.19623 | 67.92553 |
| 60.89978 | 53.33395 |
| 29.30016 | 33.42425 |
| 29.05901 | 39.77333 |
| 34.1911 | 53.33395 |
| 18.18271 | 23.66304 |
| 29.05517 | 27.6545 |
| 20.11498 | 19.18854 |
| 35.95906 | 56.65719 |
| 63.05268 | 15.18504 |
| 83.97892 | 22.43425 |
| 37.08693 | 23.66304 |
| 40.2667 | 31.66795 |
| 34.62319 | 25.70195 |
| 29.88482 | 28.99476 |
| 27.80102 | 104.78808 |
| 27.63465 | 31.66795 |
| 35.20017 | 136.85167 |
| 29.47782 | 60.08076 |
| 67.10271 | 68.72832 |
| 17.70737 | 108.27557 |
| 32.15276 | 74.62576 |
| 19.93771 | 117.22667 |
| 34.53254 | 53.33395 |
| 24.13231 | 39.77333 |
| 31.89253 | 57.89532 |
| 22.28842 | 27.6545 |
| 59.27325 | 68.72832 |

| | |
|---|---|
| 22.5821 | 27.6545 |
| 27.19797 | 38.26195 |
| 28.67452 | 88.53858 |
| 16.79395 | 28.99476 |
| 52.41833 | 39.77333 |
| 26.6669 | 32.31583 |
| 18.98107 | 62.89001 |
| 21.76655 | 19.18854 |
| 37.58305 | 122.45058 |
| 19.29699 | 101.265 |
| 53.62427 | 35.70651 |
| 35.72114 | 32.31583 |
| 20.72409 | 20.36395 |
| 39.33138 | 86.54625 |
| 29.07148 | 61.99825 |
| 14.91247 | 41.1374 |
| 31.14355 | 25.70195 |
| 36.83725 | 374.11792 |
| 28.19676 | 28.99476 |
| 41.20137 | 64.94357 |
| 32.07451 | 25.70195 |
| 118.12907 | 39.7435 |
| 54.2091 | 39.77333 |
| 36.03319 | 117.22667 |
| 35.26411 | 48.01583 |
| 73.9008 | 35.70651 |
| 58.44428 | 68.17254 |
| 52.7211 | 31.66795 |
| 29.9435 | 31.66795 |
| 31.99453 | 31.66795 |
| 29.57598 | 39.77333 |
| 16.30787 | 39.05532 |
| 19.6547 | 22.98951 |
| 30.21983 | 31.04832 |
| 32.80867 | 24.75157 |
| 16.34214 | 82.68667 |
| 35.26411 | 22.43425 |
| 73.9008 | 53.10054 |
| 58.44428 | 39.77333 |
| 52.7211 | 226.89221 |
| 29.9435 | 92.57557 |
| 31.99453 | 48.00432 |
| 29.57598 | 57.89532 |
| 16.30787 | 78.16454 |
| 19.6547 | 35.70651 |
| 30.21983 | 19.75583 |
| 32.80867 | 25.64333 |
| 16.34214 | 20.36395 |
| 34.0595 | 91.92245 |

| | | |
|---|---|---|
| 25.34029 | | 39.05532 |
| 19.01039 | | 28.99476 |
| 62.85164 | | 45.94553 |
| 33.78972 | | 96.6424 |
| 9.49489 | | 85.61158 |
| 63.50314 | | 22.98951 |
| 19.72625 | | 21.85231 |
| 33.55446 | | 272.67603 |
| 53.20621 | | 212.73709 |
| 23.83825 | | 131.88 |
| 33.24815 | | 33.42425 |
| 56.66925 | | 20.36395 |
| 24.6763 | | 31.66795 |
| 55.92166 | | 25.64333 |
| 73.11837 | | 28.32856 |
| 58.55009 | | 52.93831 |
| 20.38509 | | 39.05532 |
| 19.43987 | | 36.60403 |
| 25.45329 | | 33.80576 |
| 20.04825 | | 52.17476 |
| 13.15852 | -- | |
| 30.67522 | | |
| 13.251 | | |
| 22.9499 | | |
| 22.18639 | | |
| 25.14084 | | |
| 22.12898 | | |
| 22.13997 | | |
| 53.86194 | | |
| 31.67276 | | |
| 53.25642 | | |
| 19.79006 | | |
| 20.59801 | | |
| 42.16366 | | |
| 22.48561 | | |
| 31.0178 | | |
| 28.5602 | | |
| 30.04496 | | |
| 28.64857 | | |
| 32.31338 | | |
| 26.42034 | | |
| 28.94697 | | |
| 51.12891 | | |
| 56.85749 | | |
| 15.97273 | | |
| 27.2958 | | |
| 75.9094 | | |
| 24.8197 | | |
| 42.52441 | | |

| |
|---|
| 23.44299 |
| 21.35013 |
| 16.73466 |
| 47.05456 |
| 61.38902 |
| 47.85622 |
| 75.53772 |
| 33.2437 |
| 46.17186 |
| 43.946 |
| 16.44616 |
| 73.89602 |
| 75.79738 |
| 20.77032 |
| 23.11383 |
| 28.09427 |
| 20.23194 |
| 52.39988 |
| 22.48357 |
| 25.54732 |
| 19.46326 |
| 33.2026 |
| 88.50591 |
| 71.38612 |
| 31.9478 |
| 14.51554 |

**A3A-free protein volume(Fig.3B), N=148**

| |
|---|
| 20.36395 |
| 25.64333 |
| 25.64333 |
| 23.66304 |
| 36.60403 |
| 136.3634 |
| 20.36395 |
| 17.7635 |
| 23.98332 |
| 25.70195 |
| 25.64333 |
| 17.69076 |
| 42.48001 |
| 33.80576 |
| 96.6424 |
| 162.90477 |
| 60.64125 |
| 25.64333 |
| 136.3634 |
| 96.6424 |
| 20.36395 |
| 78.13157 |
| 25.70195 |
| 98.65514 |
| 92.57557 |
| 83.30839 |
| 60.08076 |
| 15.18504 |
| 320.83159 |
| 57.89532 |
| 45.94553 |
| 28.99476 |
| 22.98951 |
| 25.70195 |
| 28.99476 |
| 51.24951 |
| 57.89532 |
| 38.26195 |
| 33.42425 |
| 22.43425 |
| 39.77333 |
| 48.01583 |
| 35.84833 |
| 39.7435 |
| 32.31583 |
| 31.66795 |
| 32.31583 |
| 49.455 |

| |
|---|
| 45.94553 |
| 28.99476 |
| 28.60854 |
| 35.70651 |
| 35.70651 |
| 27.6545 |
| 52.17476 |
| 91.92245 |
| 56.38603 |
| 22.98951 |
| 23.66304 |
| 39.77333 |
| 35.70651 |
| 22.43425 |
| 39.77333 |
| 31.66795 |
| 39.77333 |
| 23.66304 |
| 48.00432 |
| 17.69076 |
| 28.99476 |
| 57.04333 |
| 32.31583 |
| 31.66795 |
| 57.04333 |
| 25.70195 |
| 25.70195 |
| 32.31583 |
| 32.31583 |
| 43.12476 |
| 52.17476 |
| 15.96167 |
| 27.6545 |
| 22.43425 |
| 28.99476 |
| 28.60854 |
| 35.70651 |
| 32.31583 |
| 64.94357 |
| 48.01583 |
| 45.94553 |
| 39.77333 |
| 73.005 |
| 104.78808 |
| 25.70195 |
| 25.70195 |
| 129.62129 |
| 31.04832 |
| 17.7635 |

| |
|---|
| 46.61225 |
| 38.26195 |
| 39.7435 |
| 19.18854 |
| 66.85583 |
| 39.7435 |
| 25.70195 |
| 60.08076 |
| 46.61225 |
| 28.60854 |
| 96.6424 |
| 35.66831 |
| 22.43425 |
| 35.70651 |
| 52.93831 |
| 88.53858 |
| 45.48395 |
| 62.89001 |
| 35.70651 |
| 39.7435 |
| 31.66795 |
| 25.64333 |
| 39.77333 |
| 35.66831 |
| 35.66831 |
| 15.65395 |
| 25.64333 |
| 25.64333 |
| 22.98951 |
| 39.77333 |
| 156.6075 |
| 43.87156 |
| 31.04832 |
| 31.66795 |
| 35.70651 |
| 39.44625 |
| 17.7635 |
| 25.70195 |
| 28.99476 |
| 78.13157 |
| 49.455 |
| 39.05532 |
| 42.48001 |
| 52.17476 |
| 35.70651 |
| 39.05532 |
| 86.54625 |
| 39.05532 |
| 25.70195 |

25.70195

**A3A-mutant-complex-volume (Fig. 7A), N=167**

| |
|---|
| 66.85583 |
| -- |
| -- |
| 78.13157 |
| 435.41333 |
| 80.56403 |
| 386.74333 |
| 80.56403 |
| 136.85167 |
| 56.38603 |
| 93.82529 |
| 121.38821 |
| 82.68667 |
| 67.92553 |
| 73.005 |
| 213.1275 |
| 104.78808 |
| 279.0675 |
| 244.92 |
| 60.64125 |
| 49.455 |
| 78.16454 |
| 350.39103 |
| 83.30839 |
| 64.94357 |
| 73.66231 |
| 281.59677 |
| 633.88122 |
| 48.00432 |
| 174.00833 |
| 66.85583 |
| 281.59677 |
| 56.38603 |
| 67.92553 |
| 78.13157 |
| 85.31956 |
| 68.15749 |
| 43.12476 |
| 39.7435 |
| 28.99476 |
| 22.43425 |
| 94.30153 |
| 126.90833 |
| 62.89001 |
| 93.82529 |
| 32.31583 |
| 129.91645 |
| 32.31583 |

| |
|---|
| 64.94357 |
| 115.60015 |
| 31.66795 |
| 180.81167 |
| 32.31583 |
| 48.01583 |
| 39.77333 |
| 20.36395 |
| 101.265 |
| 45.94553 |
| 99.17167 |
| 39.77333 |
| 45.48395 |
| 45.48395 |
| 32.31583 |
| 45.94553 |
| 205.32303 |
| 121.21656 |
| 91.92245 |
| 51.24951 |
| 52.17476 |
| 39.77333 |
| 20.36395 |
| 22.98951 |
| 141.52608 |
| 62.89001 |
| 63.99529 |
| 48.01583 |
| 63.99529 |
| 87.65833 |
| 61.99825 |
| 87.65833 |
| 346.185 |
| 88.53858 |
| 48.01583 |
| 183.69 |
| 198.01259 |
| 33.42425 |
| 66.85583 |
| 51.24951 |
| 25.70195 |
| 78.16454 |
| 115.60015 |
| 51.24951 |
| 176.67158 |
| 39.77333 |
| 45.27514 |
| 45.94553 |
| 67.92553 |

| |
|---|
| 25.64333 |
| 132.67861 |
| 36.60403 |
| 42.33557 |
| 39.05532 |
| 87.65833 |
| 13.64225 |
| 49.455 |
| 121.38821 |
| 121.38821 |
| 62.89001 |
| 22.43425 |
| 109.5075 |
| 52.17476 |
| 181.66261 |
| 117.22667 |
| 66.85583 |
| 52.17476 |
| 64.94357 |
| 132.67861 |
| 91.92245 |
| 70.97708 |
| 35.26639 |
| 71.61084 |
| 71.61084 |
| 64.94357 |
| 88.53858 |
| 92.57557 |
| 156.6075 |
| 31.04832 |
| 213.1275 |
| 129.91645 |
| 45.94553 |
| 60.64125 |
| 32.31583 |
| 42.48001 |
| 53.01157 |
| 31.04832 |
| 69.29614 |
| 52.17476 |
| 82.68667 |
| 142.74859 |
| 37.67058 |
| 39.77333 |
| 43.87156 |
| 69.29614 |
| 25.64333 |
| 25.64333 |
| 31.66795 |

| |
|---|
| 67.77167 |
| 111.24811 |
| 32.31583 |
| 57.59859 |
| 39.44625 |
| 52.93831 |
| 39.77333 |
| 35.70651 |
| 129.62129 |
| 60.64125 |
| 60.83645 |
| 56.61839 |
| 20.36395 |
| 132.67861 |
| 82.68667 |
| 341.21333 |
| 80.56403 |
| 101.43456 |
| 96.6424 |
| 87.27159 |

**A3A-mutant-free protein volume ( Fig. 7B), N=140**

| |
|---|
| 53.01157 |
| 22.98951 |
| 14.24448 |
| 28.99476 |
| 11.65254 |
| 39.77333 |
| 19.18854 |
| 5.68549 |
| 11.65254 |
| 15.18504 |
| 13.64225 |
| 25.64333 |
| 104.58084 |
| 25.64333 |
| 17.7635 |
| 52.93831 |
| 117.22667 |
| 13.64225 |
| 28.60854 |
| 31.66795 |
| 13.64225 |
| 48.00432 |
| 60.64125 |
| 15.65395 |
| 22.43425 |
| 129.91645 |
| 19.18854 |
| 17.7635 |
| 17.7635 |
| 32.31583 |
| 19.18854 |
| 73.66231 |
| 48.01583 |
| 17.7635 |
| 17.7635 |
| 56.38603 |
| 35.70651 |
| 32.31583 |
| 25.70195 |
| 104.78808 |
| 39.77333 |
| 22.98951 |
| 67.92553 |
| 17.7635 |
| 42.48001 |
| 60.64125 |
| 38.26195 |
| 35.70651 |

| |
|---|
| 27.6545 |
| 23.66304 |
| 23.66304 |
| 34.02504 |
| 9.68167 |
| 11.65254 |
| 62.89001 |
| 48.01583 |
| 39.05532 |
| 17.7635 |
| 64.94357 |
| 48.01583 |
| 17.7635 |
| 22.98951 |
| 32.31583 |
| 39.05532 |
| 17.7635 |
| 32.31583 |
| 60.64125 |
| 32.31583 |
| 45.94553 |
| 52.17476 |
| 53.01157 |
| 15.65395 |
| 25.64333 |
| 226.89221 |
| 48.00432 |
| 27.6545 |
| 15.18504 |
| 10.0705 |
| 39.77333 |
| 19.18854 |
| 32.31583 |
| 136.85167 |
| 129.62129 |
| 48.01583 |
| 13.64225 |
| 39.05532 |
| 28.99476 |
| 39.77333 |
| 28.32856 |
| 33.42425 |
| 17.69076 |
| 35.70651 |
| 78.13157 |
| 19.18854 |
| 39.77333 |
| 57.04333 |
| 56.38603 |

52.93831
62.89001
28.99476
68.72832
52.93831
31.66795
110.97754
110.97754
35.70651
17.7635
11.65254
15.18504
15.18504
78.13157
25.70195
19.18854
209.21611
141.52608
32.31583
31.66795
22.98951
20.36395
73.005
52.93831
22.98951
39.77333
31.66795
68.72832
43.87156
19.75583
48.00432
27.6545
136.85167
15.65395
39.05532
73.70208
86.54625
52.17476
60.08076
115.33639
25.70195
39.77333
